# Human influence and biotic homogenization drive the distribution of *Escherichia coli* virulence genes in natural habitats

**DOI:** 10.1002/mbo3.445

**Published:** 2017-02-18

**Authors:** Adriana Cabal, Joaquin Vicente, Julio Alvarez, Jose Angel Barasona, Mariana Boadella, Lucas Dominguez, Christian Gortazar

**Affiliations:** ^1^ VISAVET Health Surveillance CentreUniversidad ComplutenseMadridSpain; ^2^ SaBio IRECNational Wildlife Research Institute (CSIC‐UCLM‐JCCM)Ciudad RealSpain; ^3^ Department of Veterinary Population MedicineCollege of Veterinary MedicineUniversity of MinnesotaSt. PaulMNUSA

**Keywords:** *Escherichia coli*, natural habitats, pathotypes, virulence genes, wildlife

## Abstract

Cattle are the main reservoirs for Shiga‐toxin‐producing *Escherichia coli* (STEC), the only known zoonotic intestinal *E. coli* pathotype. However, there are other intestinal pathotypes that can cause disease in humans, whose presence has been seldom investigated. Thus, our aim was to identify the effects of anthropic pressure and of wild and domestic ungulate abundance on the distribution and diversity of the main human *E. coli* pathotypes and nine of their representative virulence genes (VGs). We used a quantitative real‐time PCR (qPCR) for the direct detection and quantification of the genus‐specific gene *uid*A, nine *E. coli* VGs (*stx1*,* sxt*2, *eae*,* ehx*A, *agg*R, *est*,* elt*,* bfp*A, *inv*A), as well as four genes related to O157:H7 (*rfb*_O157_, *fli*C_H7_) and O104:H4 (*wzx*_O104_, *fli*C_H4_) serotypes in animals (feces from deer, cattle, and wild boar) and water samples collected in three areas of Doñana National Park (DNP), Spain. Eight of the nine VGs were detected, being *inv*A, *eae*, and *stx*2 followed by *stx*1, *agg*R, and *ehx*A the most abundant ones. In quantitative terms (gene copies per mg of sample), *stx*1 and *stx*2 gave the highest values. Significant differences were seen regarding VGs in the three animal species in the three sampled areas. The serotype‐related genes were found in all but one sample types. In general, VGs were more diverse and abundant in the northern part of the Park, where the surface waters are more contaminated by human waste and farms. In the current study, we demonstrated that human influence is more relevant than host species in shaping the *E. coli* VGs spatial pattern and diversity in DNP. In addition, wildlife could be potential reservoirs for other pathotypes different from STEC, however further isolation steps would be needed to completely characterize those *E. coli*.

## 1 Introduction

A large number of infectious agents, including those most important to the microbiological safety of food and water, have been identified in domestic animals and wildlife. Food‐borne bacterial pathogens evolve in response to environmental changes, developing new virulence properties and occupying new niches (Newell et al., 2010). Bacterial pathogens acquired their pathogenic capability by incorporating different genetic elements through horizontal gene transfer (Koonin, Makarova, & Aravind, 2001) and thus the ancestors of virulent bacteria, as well as the origin of virulence determinants, lay most likely in the environmental microbiota (Martinez, 2013). The ubiquitously distributed enterobacterium *Escherichia coli* (E. coli) is naturally present in the lower intestinal tracts of humans and warm‐blooded animals. *E. coli* can survive for long time in the environment, where so‐called “naturalized” populations may coexist with strains of vertebrate origin (Ishii & Sadowsky, 2008). *E. coli* genotypes present in ecosystems are also influenced by environmental factors such as temperature and hydrology, and by anthropogenic factors that include the proximity to urban areas and livestock production systems, with higher numbers and a greater diversity of *E. coli* genotypes closer to settlements and farms (Lyautey et al., 2010). The risks for Public Health posed by livestock and wild animals carrying pathogenic *E. coli* are dependent on the prevalence, incidence, and magnitude of pathogen carriage in the animal hosts, and the degree of interaction between the animals and humans (Jay et al., 2007). Ungulate animals are among the most common reservoir species for Shiga‐toxigenic *E. coli* (STEC), a zoonotic pathotype for which cattle are considered the main reservoirs (Hancock, Besser, Lejeune, Davis, & Rice, 2001). In addition, *E. coli* O157:H7 and other non‐O157 STEC are present in a large variety of other ungulates such as deer, sheep, goats, or pigs (Doane et al., 2007). With regard to wildlife, the most abundant species in a particular region would be the most likely concern in terms of pathogen shedding since the risk of fecal contamination by these animals is the highest. In studies on free‐ranging deer, the fecal prevalence of *E. coli* O157:H7 was estimated to range from zero to less than 3% (Branham, Carr, Scott, & Callaway, 2005; Dunn, Keen, Moreland, & Alex, 2004; Fischer et al., 2001; Renter, Sargeant, Hygnstorm, Hoffman, & Gillespie, 2001; Sargeant, Hafer, Gillespie, Oberst, & Flood, 1999), while in feral pigs, 23% of fecal samples were positive for *E. coli* O157 in California, USA (Branham et al., 2005).

Until now, some studies for detection of STEC in large game animals such as the red deer (*Cervus elaphus*) or the Eurasian wild boar (*Sus scrofa*) have been developed (Miko et al., 2009; Sanchez et al., 2009). However, fewer studies have investigated other *E. coli* intestinal pathotypes (EPEC = enteropathogenic *E. coli*, ETEC = enterotoxigenic *E. coli*, EIEC = enteroinvasive *E. coli*, EAEC = enteroaggregative *E. coli*) in wild ungulates (Chandran & Mazumder, 2013; Li et al., 2013), and thus there is a lack of epidemiological data regarding their distribution, which would be especially relevant at the wildlife/livestock/human interface.

Using a set of quantitative real‐time PCRs (qPCRs) for the direct detection and quantification of nine *E. coli* virulence genes (VGs), we used Doñana National Park (DNP) as a natural experiment to identify the effects of anthropic pressure and of wild and domestic ungulate abundance on the distribution and abundance of human pathogenic *E. coli* genotypes and VGs. We expect that higher interspecies transmission of *E. coli* may arise from increased ecological overlap (Barasona et al., 2014; Barasona et al., 2015; Goldberg, Gillespie, Rwego, Estoff, & Chapman, 2008), and that the spatial pattern of distribution of pathogenic VGs in the environment and hosts may be affected by human, livestock, and wildlife distribution. We hypothesized that *E. coli* VGs would be more diverse and abundant in proximity to human settlements and waste than in natural habitats, with human influence being more relevant than host species in shaping their spatial pattern.

## 2 Methods

### 2.1 Study area

DNP (37°0′ N, 6°30′ W, covering an area of approximately 54,000 ha with the highest level of environmental protection in Spain), located in the south‐west Iberian Peninsula, is considered one of the most important European wetlands in terms of biodiversity. This is a flat region of sandy soils, with altitudes ranging from 60 m above sea level (asl) to 0 m asl in the south marshland area. It contains the largest wetland in Western Europe, an intricate matrix of marshlands (270 km^2^). Natural inundation takes place between October and March, mostly by rain in the drainage watershed. Under natural conditions, most of the contributions of water come from precipitation, streams in the north‐west (La Rocina, El Partido, Las Cañadas, which is included in our study area), and rivers in the east (Guadalquivir and Guadiamar, which are now diverted, entries occurring through the Guadalquivir estuary in the east, outside our study area) (Aldaya, García‐Novo, & Llamas, 2010). Traditional farming is being progressively abandoned, and greenhouse farming and rice paddies have become the most productive activities around DNP, together with touristic resorts (Haberl et al., 2009).

**Figure 1:**
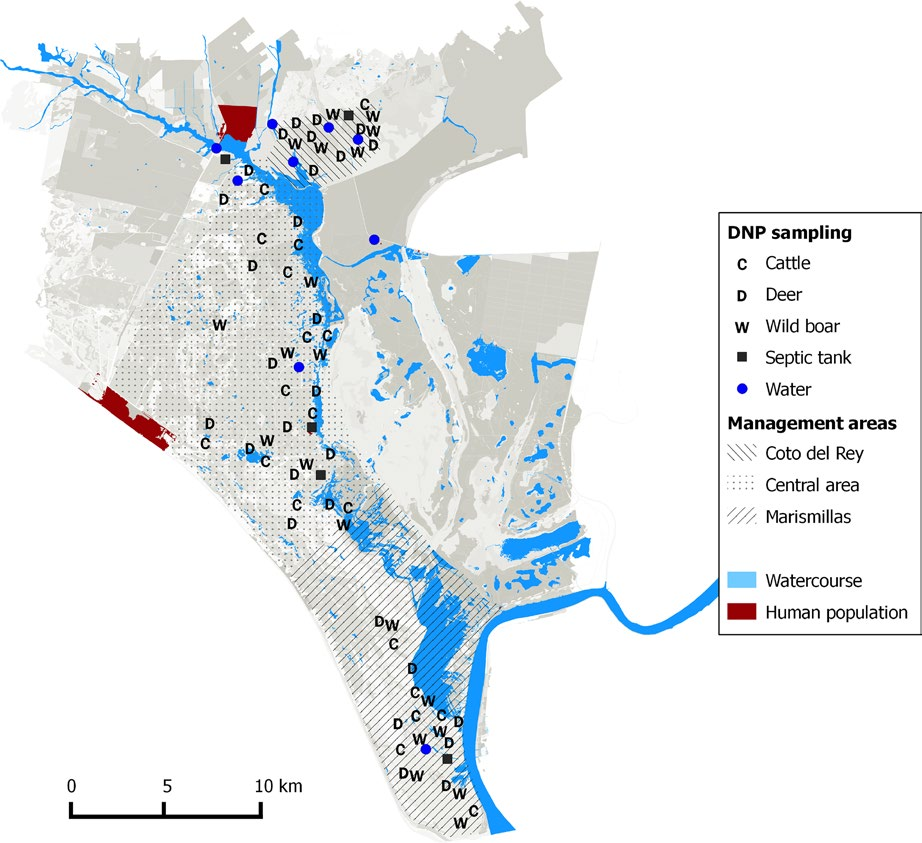
Map of Doñana National Park (DNP) and surroundings. Environmental features, sampling type, sites, and areas are shown. Watercourses in the north represent the entrance of water from outside the park. The habitat east to the three study areas is composed by marsh

Aside from the temporary marshland, DNP has a large number of small, more or less permanent water bodies and watercourses (Figure 1). Some streams flow from the higher regions in the north‐west and drain southward into the marshland. These streams have not significantly improved their water quality in the last two decades despite the construction of waste water treatment plants (Serrano et al., 2006). DNP has a mediterranean climate generally classified as dry subhumid with marked seasons. In the wet season (winter and spring), the marshland is flooded, and wild and domestic ungulates graze in the more elevated scrublands. The hardest season for ungulates in DNP is summer (from July to September), when herbaceous vegetation, wetlands, and water bodies in most habitats dry up and only a few meadows remain green at the ecotone between the upper scrublands and the lower marshes (Braza & Alvarez, 1987). DNP represents a unique setting where wildlife and cattle share habitat with a proximity gradient to human settlements toward the park boundary. Local variation in wildlife abundance and cattle distribution, along with the seasonally increased aggregation of livestock and wildlife at water points, makes DNP ideal for research on indirectly transmitted disease agents (Green & Silverman, 1994).

Three different cattle areas from north to south can be identified in DNP (Figure 1). Coto del Rey (CdR) is in the north border where cattle are absent. In the central area, the biological reserve and its surroundings Estación Biológica Doñana (EBD) includes three cattle enclosures (*n* = 670 cattle in total; average density = 4.2 cattle/km^2^). Marismillas (MAR) is the south of DNP (*n* = 318 cattle; density = 3.1 cattle/km^2^). Wild ungulates present in DNP are the Eurasian wild boar (*S. scrofa*), red deer (*C. elaphus*), and fallow deer (*Dama dama*). Data on their abundance and water use by ungulates were provided by mammal monitoring services in Estación Biológica Doñana (CSIC, http://www-rbd.ebd.csic.es/Seguimiento/seguimiento.htm) and by camera trapping surveys (Barasona et al., 2016), respectively.

### 2.2 Sample collection

A survey was carried out during June–September 2012, when water availability is critical, and therefore livestock and wild ungulates aggregate more around water sites. The sampling strategy was designed to represent the north (where water from the streams pours into the marshes) to south (dry dune habitats) gradient of DNP, and the east to west gradient (from the marsh to the woodlands). Collection of samples was performed using disposable sterile material and containers, and sampling sites were georeferenced by Global Positioning System. We collected 14 water samples (variable volume), nine from surface water (creeks and waterholes) and five from septic tanks using sterile containers. We also collected 68 pooled fresh fecal samples from the ground (from 3 to 7 individual fecal samples per pool) from either red deer or fallow deer (29 pools, *n* = 148 fecal samples), wild boar (20 pools, *n* = 92 fecal samples), and cattle (19 pools, *n* = 87 fecal samples) in sterile plastic bags (Figure 1). All samples were sent for refrigeration on the same day to the laboratory and immediately frozen upon arrival for further analysis.

### 2.3 Laboratory analyses

Water samples and pooled fecal samples were processed and analyzed by using a previously described qPCR assay in order to detect a set of nine VGs (see Table S1) characteristic of different *E. coli* enteric pathotypes (*stx*1, *stx*2, *eae*,* Inv*A, *ehx*A, *est*,* elt*,* bfp*A, *agg*R), four serotype‐related genes (*rfb*_O157_, *fli*C_H7_, *wzx*_O104_, *fli*C_H4_), and one genus‐specific gene (*uid*A) (Cabal et al., 2013; Cabal et al., 2015). Pooled fecal samples were processed in a 1/3 proportion of phosphate‐buffered saline. Briefly, 400 mg of each pool of feces were used for DNA extraction with a commercial kit (QIAamp DNA stool mini‐kit, Qiagen, Hilden, Germany) and extracted DNA was directly used in the qPCR. Water samples were concentrated by double centrifugation at 16 Relative centrifugal force during 15 min. Supernatants were then mixed together with the sediment to get a final volume of 400 μl per sample. Then, DNA was extracted using the same commercial kit. Finally, qPCRs were performed as described previously (Cabal et al., 2015; Cabal et al., 2015).

### 2.4 Statistics

Kruskal–Wallis and Mann–Whitney *U* nonparametric tests were used to compare the number of *uid*A copies per mg of feces, considered as an indicator of the overall *E. coli* load in each sample, among hosts species and zones.

Proportion of positive samples to certain VG combinations depending on the sample type was evaluated using Fisher's exact test.

Explanatory covariates were determined following the revision of the landscape and animal factors regulating *E. coli* presence, and based on the accessible information for DNP, we selected 16 potential predictors (see Table S2), derived from a geographic information system (GIS) of the study area using Quantum GIS version 1.8.0 Lisboa (QGIS Development Team, 2012). In a first step, we screened against including collinear covariates using a |r| = .6 as a threshold cut‐off value (Hosmer & Lemeshow 2000). As a result, in a second step the noncollinear variables in the previous step were included as explanatory ones in generalized linear models (GLMs): host species, distance to nearest surface water entrance to DNP, riparian habitat proportion, distance to nearest permanent water point, distance to nearest marsh–shrub humid ecotone, ungulate abundance (per sampling area), and water conservation status (in the nearest water point), respectively, for each VG and host (wild boar, deer, and cattle) (Green & Silverman, 1994). In this second step, we tested the final predictors affecting the presence of *E. coli* VGs using a binomial error (0 = negative, 1 = positive) and a logic link function. Distances, abundances, water status, and land cover type proportions were treated as continuous variables, while host species as a categorical variable (see Table S2). Regarding the VG diversity (defined as the number of different VGs present, ranging from 1 to 8), we used a Poisson error and an identity link function. All statistics were performed in SPSS Statistics 18 for Windows (IBM^®^, Armonk, NY, USA).

## 3 Results

### 3.1 Descriptive epidemiology

**Figure 2:**
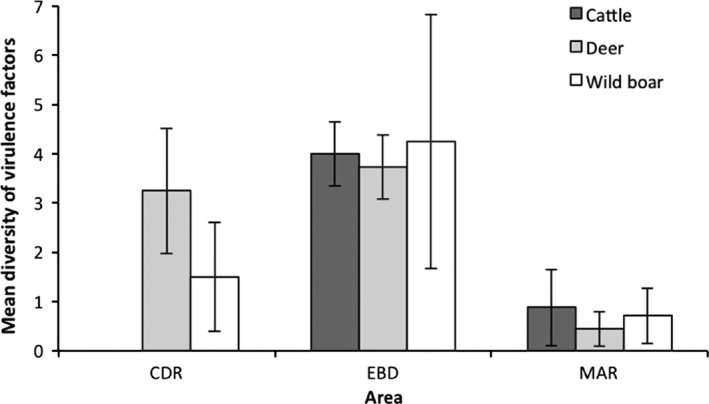
Mean number of virulence genes (VGs, average diversity and _95% C.I._) detected in cattle, deer, and wild boar depending on the park zone (North Coto del Rey [CDR], Central EBD, South Marismillas [MAR])

**Figure 3:**
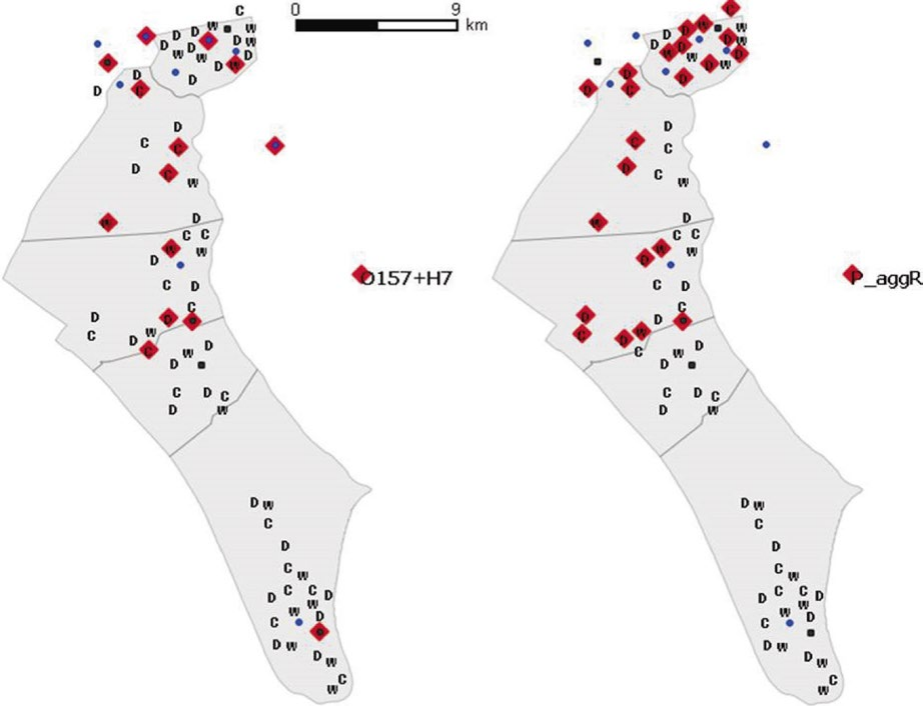
Spatial distribution of selected *Escherichia coli* genes (characteristic genes *rfb*_O157_, *fli*C_H7_, and virulence gene [VG] *agg*R) detected in pooled fecal samples (C: cattle; D: deer; W: wild boar). Surface water (blue dots) and septic tanks (black squares) collected in Doñana National Park (DNP), Spain. Positive samples are indicated in red

All samples, but one cattle pool (18/19, 94.7%), one deer pool (28/29, 96.5%), and one wild boar pool (19/20, 95%), tested positive for the genus‐specific gene *uid*A, including all nine surface water and all five septic tank samples (Table 1). The mean number of *uid*A copies per mg of sample is shown in Table 2. Statistical differences in the number of *uid*A copies were observed depending on the type of sample, with higher values in environmental than in animal samples (Mann–Whitney *U* test, *p < *.05). No statistical differences were evidenced when comparing septic tanks against surface waters (Mann–Whitney *U* test, *p *=* *.79). Differences depending on the type of sample, species, and zones for VGs are shown in Table 2. The number of positive samples to each VG varied largely depending on sample, host species, and area (Table 1), and in some cases, qualitative values differed from quantitative ones (Table 2). Overall, the pattern of VG diversity was decreasing from north to south, and decreased particularly in the southernmost part of the park, with little anthropogenic influence (MAR area, Figure 2, Kruskal–Wallis tests statistically significant, *p *<* *.05 for the three host species, respectively). The qualitative analyses revealed that the EIEC‐associated VG (*inv*A) was the most abundant gene in all samples/areas (45/82), followed by *eae* (41/82) and *stx*2 (36/82), which were also frequently detected. *Ehx*A (21/82), *stx*1 (24/82), and *agg*R (22/82) were moderately detected (Figure 3). The STEC‐associated VGs (*stx*1, *stx*2, *ehx*A, and *eae*) were present in all combinations samples/hosts in at least one of the areas sampled. On the contrary, ETEC and typical EPEC‐associated VGs (*est*,* elt*, and *bfp*A) were absent or present in very few samples (Table 1). All VGs were found in deer and wild boar samples. *Inv*A and *stx*2 were the most frequently detected VGs in ruminants, followed by *eae* (deer and cattle) and *agg*R (deer), while in wild boar the most frequently found genes were *eae* and *inv*A. Two VGs, *eae* and *ehx*A, were often detected in septic tanks, and *inv*A was most frequent and abundant in superficial water. Interestingly, *est* was present in wild ungulates but absent in cattle and water samples. The VGs *agg*R and *est* were not detected in the southern third of DNP, further away from anthropogenic influences (Table 1).

**Table 1:** Positive samples to each *E. coli* gene in pooled fecal samples and environmental samples collected in Doñana National Park (DNP) in June–October 2012

Source	Total	*uid*A	*stx*1	*stx*2	*eae*	*agg*R	*st*	*ehx*A	*bfp*A	*inv*A	Mean Div.^a^	SD Div.^a^	*rfb*_O157_/*fli*C_H7_	STEC^b^	*wzx*_O104_/*fli*C_H4_
Cattle	**19**	**18**	6	12	9	4	0	6	1	10	2.526	1.896	4	3	1
MAR	8	7	1	3	1	0	0	0	1	1	0.875	1.126	0	0	0
CdR	1a	1	0	0	0	1	0	0	0	0	1	—	0	0	0
EBD	10	10	5	9	8	3	0	6	0	9	4	1.054	4	3	1
Deer	**29**	**28**	8	15	14	12	3	2	1	18	2.517	1.864	1	0	3
MAR	9	9	1	2	0	0	0	0	0	1	0.44	0.527	0	0	1
CdR	8	7	1	3	6	6	2	0	1	7	3.25	1.832	0	0	2
EBD	12	12	6	10	8	6	1	2	0	10	3.583	1.165	1	0	0
Septic tank	**5**	**5**	1	1	4	1	0	3	1	2	2.6	2.074	3	1	0
MAR	1	1	0	0	1	0	0	1	0	1	3	—	1	0	0
CdR	1	1	0	0	1	0	0	0	0	0	1	—	0	0	0
EBD	3	3	1	1	2	1	0	2	1	1	3	2.646	2	1	0
Water	**9**	**9**	6	5	4	0	0	5	0	6	2.889	1.616	3	1	1
MAR	1	1	0	0	0	0	0	0	0	1	1	—	0	0	0
CdR	5	5	4	3	3	0	0	5	0	4	3.8	0.837	3	1	0
EBD	3	3	2	2	1	0	0	0	0	1	2	2	0	0	1
Wild boar	**20**	**19**	3	3	10	5	1	5	5	9	2.05	2.259	3	1	1
MAR	8	7	0	0	3	0	0	1	0	1	0.625	0.744	0	0	1
CdR	6	6	0	0	2	2	0	1	2	2	1.5	1.378	1	0	0
EBD	6	6	3	3	5	3	1	3	3	6	4.5	2.429	2	1	0
Total	**82**	**79**	**24**	**36**	**41**	**22**	**4**	**21**	**8**	**45**	2.451	1.932	14	6	6

CdR, Coto del Rey; MAR, Marismillas.

A single cattle sample was collected outside, but bordering CDR.

a Data are referred to the diversity of VGs.

b Referred to the combination of STEC‐associated VGs.

**Table 2:** Mean quantitative values (gene per mg of feces) for each VG per zone and sample origin

Origin	*uid*A	*stx*1^b^	*stx*2^b^ ^c^	*eae*	*agg*R	*est*	*ehx*A^b^	*bfp*A	*inv*A
Cattle	12,882.2	879.5	2,991.1^a^	100.5^a^	81.4	0	66.9^a^	0.27	760.5^a^
MAR	413.3	153.3	369.2	0.21	0	0	0	0.27	1.6
CdR	35.6	0	0	0	0.22	0	0	0	0
EBD	12,433.3	726.1	2,621.9	100.3	81.2	0	66.9	0	758.9
Deer	291,896.9	6,279.2	2,081,743.4	175.9^a^	260.9^a^	16,579.9	854.2	0.76	234.7^a^
MAR	10,027.5	2,466	10,421.3	0	0	0	0	0	0.84
CdR	204,272.2	43.7	2,066,022	102.4	4.17	16,504.2	0	0.76	91.5
EBD	77,597.1	3,769.5	5,300.1	73.5	256.8	75.7	854.2	0	142.3
Septic tank	247,430.1	1,620	181.5	1,407.6	17.5	0	341.1	69.7	1,051.9
MAR	5,430	0	0	537	0	0	16.1	0	141.4
CdR	49.3	0	0	30.6	0	0	0	0	0
EBD	241,950.8	1,620	181.5	840	17.5	0	325	69.7	910.5
Surface water	147,019.7	112,302	22,053.6	621.5	0	0	506.6	0	13,047.3
MAR	273	0	0	0	0	0	0	0	23.5
CdR	34,389	16,617	4,168.5	558.9	0	0	506.6	0	12,849.7
EBD	112,357.7	95,685	17,885.1	62.5	0	0	0	0	174
Wild boar	127,537.9	3,964.3^a^	709.6^a^	74.6	187.05	15.3	1,086.4	31.9	114.6^a^
MAR	47,888.8	0	0	34.1	0	0	86.2	0	3.9
CdR	3,395.5	0	0	6.4	0.75	0	2.80	30.8	10.6
EBD	76,253.6	3,964.3	709.6	34	186.3	15.3	997.3	1.11	100.09
Total^d^	826,767.14	125,045.08	2,107,679.4	2,380.3044	547.0704	16,595.28	2,855.4515	102.753	15,209.2488

CdR, Coto del Rey; MAR, Marismillas.

a Statistically significant differences among study sites for each VG and type of sample (Kruskal–Wallis test).

b Statistically significant differences among type of sample for each VG (Kruskal–Wallis test).

c Statistically significant differences among species for each VG (Kruskal–Wallis test).

d Sum of the mean values obtained for each matrix.

The serotype‐related genes *rfb*_O157_ and *fli*C_H7_ were detected simultaneously in 4 (21.1%) of 19 cattle samples, 1 (3.4%) of 29 deer samples, and 3 (15.0%) of 20 wild boar samples. In contrast, these genes were detected in three of five septic tanks and in three of nine surface water samples. However, samples positive to *rfb*_O157_/*fli*C_H7_ that were also positive to STEC typical VGs were even less frequent (cattle: 15.8%, wild boar: 5%, and deer: 0%). In the southern third of DNP this combination was only found in a septic tank (Figure 3). The probability of detection for this combination was higher in water samples (either superficial or septic tank) than in animal samples (6/14 vs. 8/68; Fisher's *p *=* *.006). The serotype‐related genes *wzx*_O104_ and *fli*C_H4_ were found together in 1 (5.3%) cattle, 3 (10.3%) deer, and 1 (5%) wild boar samples. This combination was not observed in septic tanks and was present in only one surface water sample. Three of these six detections corresponded to the northernmost sampling sites in DNP. One of the deer samples also carried *agg*R, but all *wzx*_O104_ and *fli*C_H4_ positive samples were negative for *stx*2.

Mean values for the quantitative presence of each VG are shown in Table 2. Briefly, the highest values were reported for *stx*1 and *stx*2 (>10^5^ gene copies per mg or ml), followed by *est* and *inv*A (>10^4^). By sample source, the highest values for cattle and deer were reported for *stx*2 (>10^3^ and >10^6^, respectively), while in wild boar, *stx*1 gave the highest mean values (>10^3^). In the septic tanks, *stx*1 and *inv*A were found at high levels (>10^3^), and for the superficial water, *stx*1 and *stx*2 gave the highest results (>10^5^).

In cattle, deer, and wild boar, significant differences were found among the study areas for some VGs. Also, significant differences were seen for *stx*1, *stx*2, and *ehx*A mean values by sample origin. Finally, significant differences were detected for *stx*2 mean values among animal species (Table 2).

### 3.2 Factors affecting the presence of *E. coli* VGs

A summary of the GLMs results for the presence of the eight individual VGs, the EHEC/STEC typical VGs (*stx*1, *stx*2, *eae*,* ehx*A), *rfb*_O157_/*fli*C_H7_ and *wzx*_O104_/*fli*C_H4_ combinations, and the total number of different VGs (diversity) as a function of host species, host abundance, and environmental variables is shown in Table 3. After controlling by other factors, no association between the presence of any VG, gene combinations, or the total number of VG detected and any particular host species was found. Models showed that the closer the sampling site was to the entrance of surface water to the park, the higher the risk of the sample to test positive for VGs *stx*2, *eae*, and *inv*A (*stx*1 was marginally significant), so as for the combination *rfb*_O157_/*fli*C_H7_. As a result, the proximity to the water entrance was statistically positively associated to an increased number of different VGs (diversity) in the fecal sample. The VG *agg*R was statistically more prevalent as distance to marsh increased. *Stx*2 and the combination *rfb*_O157_/*fli*C_H7_ were statistically more frequent at higher abundances of ungulates.

**Table 3:** *P*‐values of the GLM for the presence of eight virulence factors, *rfb*_O157_/*fli*C_H7_, EHEC/STEC, *wzx*_O104_/*fli*C_H4_, respectively, and the diversity of different virulence factors as a function of host species, host abundance, and environmental variables

	*stx*1	*stx*2	*eae*	*agg*R	*est*	*ehx*A	*bfp*A	*Inv*A	*rfb*_O157_/*fli*C_H7_	EHEC/STEC	*wzx*_O104_/*fli*C_H4_	Diversity
Host spp.	.65	.09	.93	.34	1.00	.15	.13	0.38	.17	.97	.72	.92
*d* surface water entrance	.06	**.02** ^(−)^	**.02** ^(−)^	.09	0.37	.15	.49	**0.00** ^(−)^	**.03** ^(−)^	.45	.95	**.00** ^(−)^
Riparian habitat	.50	.69	.21	.81	0.70	.38	.48	1.00	.87	.31	.21	.59
*d* to water point	.11	.59	.38	.19	0.48	.59	.90	0.23	.17	.23	.87	.25
*d* to marsh	.26	.15	.84	**.03** ^(+)^	0.78	.18	.25	0.33	.98	.71	.69	.32
Ungulate abundance	.26	.06^(+)^ a	.63	.59	0.60	.16	.78	0.66	**.03** ^(+)^	.98	.79	.13

Statistically significant relationships (*p *<* *.05) are highlighted in bold. Associated positive parameter estimates with distance (*d*) or abundance of hosts are indicated with ^(+)^ and negative with ^(−)^.

a Marginally significant.

## 4 Discussion

Animals are considered the main source of certain pathogenic *E. coli* strains (mainly STEC strains), while humans constitute the only known reservoir of all the other pathotypes (Nataro & Kaper, 1998). For this reason, most studies performed on animal samples have focused mainly on the detection of STEC, and especially O157:H7 (Miko et al., 2009; Sanchez et al., 2009). However, animals can also host other pathotypes and VGs that could eventually lead to the emergence of new strains such as the EHEC/EAEC O104:H4 causing the German outbreak in 2011 (Bielaszewska et al., 2011; Nyholm et al., 2015), even though the origin of these strains remains unclear. For this reason, here we evaluated the presence of VGs characteristic of the intestinal pathotypes of *E. coli* in samples from livestock, wildlife, and the environment collected in different epidemiological settings in terms of anthropogenic contamination. This qPCR proved to be a fast and reliable tool to assess the frequency and quantity of each VG present in both water and fecal samples and an alternative to time‐consuming methods such as traditional bacteriology, which has been regarded as less suitable for characterization of a whole *E. coli* population (Lleo et al., 2005). Even though the simultaneous detection of a given set of VGs in a pooled fecal sample does not imply its presence in a single *E. coli* strain, its quantification provides evidence of situations in which horizontal gene transfer would be more likely to occur, potentially leading to the emergence of new pathogenic strains. According to our *uid*A results, all studied host species (cattle, deer, and wild boar) contribute to *E. coli* maintenance in DNP and to environmental contamination, at similar rates. In addition, proportion of positive samples to each molecular target varied largely depending on sample, host species, and area (Table 1). This means that while the contribution of each host species to *E. coli* maintenance is more or less uniform, its relationship with different VGs is variable. Data generally confirmed the initial hypothesis that *E. coli* VGs would be more diverse and abundant in proximity to human settlements and waste than in natural habitats. Anthropic drivers were more influential than host species in shaping *E. coli* VG spatial pattern and VG diversity, and its abundance was in general higher in the northern part of DNP closer to the human influence, while some VGs (*agg*R and *est*) were not detected in the southern third of DNP, away from anthropogenic influences.

All these findings indicate that water, both surface water and the effluents from human dwellings, plays a key role in *E. coli* epidemiology in DNP. Several studies have been performed for detection and quantification of *E. coli* in water for human consumption or agricultural and recreational uses (Khan et al., 2007), but information on the total *E. coli* numbers or its VGs in wastewater before treatment or nonpotable water in natural parks is scarce. Our results confirm that human waste may be an important source of microbial exposure to livestock and wildlife through water, and subsequent high levels of antimicrobial resistance, even within protected areas (Pesapane, Ponder, & Alexander, 2013).

Although the limited sample size of water samples prevents the extraction of definitive conclusions, other studies have also reported the presence of certain VGs typical of human pathotypes in strains recovered from surface waters, and suggest the wide spread of potentially pathogenic isolates in aquatic ecosystems. The high prevalence of positive samples to different VGs compared with other culture‐based studies performed on water samples (Carlos et al., 2011; Ramirez Castillo et al., 2013) is not surprising given the higher sensitivity of direct‐detection approaches in comparison with culture‐based techniques (Khan et al., 2007).

For instance, prevalence of the O157:H7 serotype in wildlife is normally low, and therefore O157:H7 positive wild animals are usually considered to be caused by the sporadic transmission from human beings and domestic animals (Ferens & Hovde, 2011). In our study, the *rfb*_O157_/*fli*C_H7_ combination was overall not abundant, but seemed more prevalent in cattle (15.8%) than in wild boar or deer (5% and 0%, respectively). Although the simultaneous presence of typical O157:H7 VGs such as *stx1*,* stx2*, and *eae* in the *rfb*_O157_/*fli*C_H7_‐positive samples suggested the presence of O157:H7 strains in these specimens, the probability of isolation of one single colony is extremely low as previously reported (Dunn et al., 2004; Renter et al., 2001; Sargeant et al., 1999). *Rfb*_O157_ and *fli*C_H7_ were detected together more often in septic tanks (60%) and surface water samples (33%) than in animal fecal pools (3.4%–21.1%). In addition, the high mean values for *stx*1 found in the septic tanks (containing wastewater of human origin) in comparison with those of *stx*2 were in agreement with the fact that human STEC strains usually carry only *stx*1 (with O157:H7 being an exception) (Guth, Prado, & Rivas, 2010). In contrast, the superficial water contained higher values for *stx*2 than the septic tanks and higher than *stx*1 in this matrix as reported previously (Sidhu, Ahmed, Hodgers, & Toze, 2013). These findings are probably due to animal contamination.

Although *agg*R was detected at low levels, animal samples with the highest values were found in the EBD sampling area. This meant that possibly animals in this area had a higher chance to acquire EAEC than animals from the other two sampling areas. In addition, only water samples collected from the septic tank located in the EBD area revealed the presence of *agg*R gene, suggesting a differential degree of exposure of the animals located in this area to EAEC. Also, watersheds may not have been much polluted with fecal contamination of human origin as *agg*R was not present. Interestingly, other authors already reported EAEC pathotype in sewage water not only from treatment plants (Carlos et al., 2011; Omar & Barnard, 2010) but also from domestic animals such as pigs, cattle, and chicken (Kagambega et al., 2012). None of the wildlife or livestock samples contained the typical VGs present in the EAEC/EHEC O104:H4 German outbreak strain (*stx*2/*agg*R/*wzx*_O104_/*fli*C_H4_). This contrasts with our previous report in which those characteristic VGs were detected simultaneously in samples from German cattle farms located near the outbreak area (Cabal et al., 2015).

Although the detection rate for *inv*A in DNP animal samples was higher than expected taking into account that EIEC is considered a human pathogen (Kaper, Nataro, & Mobley, 2004), the mean values (~10^2^ gene copies per mg) obtained in the quantitative analysis for this VG could indicate that it was present at low quantities in animals. In contrast, higher mean values were found in water samples (10^3^–10^4^ gene copies per ml). Presence of EIEC markers such as *inv*A may indicate the pollution of surface waters and animal foraging grounds with human feces (Cabal et al., 2015; Sidhu et al., 2013). *Shigella*, which shares the same genetic background as EIEC, has not been detected in animals. This suggests that the positive samples for *inv*A in the current study are most likely linked to EIEC.

Similarly to *inv*A, the low mean values (or the absence) found for the typical EPEC gene *bfpA*, suggested a limited degree of human fecal contamination with this pathotype. Animals have been described as reservoirs of atypical EPEC together with humans (Moura et al., 2009). Thus, the *eae* mean values seen in the septic tank together with the absence of *bfp*A could also indicate the presence of this pathotype, as *eae* is a common gene in STEC and in EPEC strains (Nataro & Kaper, 1998).

Finally, ETEC markers were detected at low frequencies although *est* in deer from CdR was high. It is possible that ETEC/STEC pathotypes carrying *est* could be also present, as seen in our previous works in cattle and other animal species (Cabal et al., 2015; Cabal et al., 2015). These hybrids have been previously described and associated to the carriage of *stx*2g (Sidhu et al., 2013).

## 5 Conclusions

Biotic homogenization is the process by which species invasions and extinctions increase the taxonomic, genetic, or functional similarity of multiple locations over a specified time interval (Olden, Leroy Poff, Douglas, Douglas, & Fausch, 2004). Results presented herein suggest that such a process is currently taking place on the *E. coli* community in DNP. As expected, given the abundant local cattle and wildlife populations, *E. coli* was detected everywhere and there were no big differences among host species or among DNP zones. However, the distribution of genes characteristic of the described pathotypes was not random. These VGs were much more prevalent in the north of DNP, close to the entry of surface waters contaminated by human settlements and farms, suggesting an effect of a closer contact with humans/livestock on the presence and abundance of VGs typical of human and livestock‐associated pathotypes. Descriptive and analytic epidemiology provided additional insights into the ecology of potentially pathogenic *E. coli* in multihost settings. However, further knowledge is needed regarding the role of animals as intermediate reservoirs for other pathotypes different from STEC.

## Conflict of Interest

All the authors declare that they have no conflict of interest in the research.
