# Supplementary material for: Human influence and biotic homogenization drive the distribution of Escherichia coli virulence genes in natural habitats
Source: Microbiologyopen. 2017 Feb 18;6(3):e00445. doi: 10.1002/mbo3.445 (PMC5458461; doi:10.1002/mbo3.445)
Supplement: Supplementary file 1 [file MBO3-6-na-s001.docx]

**SUPPLEMENTARY MATERIAL**

**Table S1. VGs used for the detection of *E. coli* intestinal pathotypes in this study.**

| **Pathotype** | **Main reservoir** | **VFs** | **VGs** |
| --- | --- | --- | --- |
| **STEC/EHEC** | Ruminants (cattle) | Shiga toxins | *stx*1 and *stx*2 |
|  |  | Intimin | *eae* |
|  |  | Enterohemolisin | *ehx*A |
| **EAEC** | Humans | Transcriptional regulator | *agg*R |
| **EPEC** | Humans | Bundilin | *bfp*A |
|  |  | Intimin | *eae* |
|  |  | Enterohemolisin | *ehx*A |
| **ETEC** | Humans | Thermostable toxin ST | *est* |
|  |  | Thermolabile toxin LT | *elt* |
| **EIEC** | Humans | Invasion protein | *inv*A |

**Table S2.** Potential explanatory covariates, descriptions, mean values (*X*) and standard deviations (SD) for the sampling sites in Doñana National Park. References are indicated.

| Code | Variable | *X* ± SD |
| --- | --- | --- |
| *d* surface water entrance | Distance to nearest surface water entrance to DNP (km) | 12.58±11.5 |
| Riparian habitat | Riparian habitat proportion (in 1 km^2^ from the sampling site centroid) (%) | 6.18±18.48 |
| *d* to water point | Distance to nearest permanent water point (km) | 0.62±0.77 |
| *d* to marsh | Distance to nearest marsh-shrub humid ecotone (km) | 0.82±1.05 |
| Water status | Water conservation status (in the nearest water point) (1-5) | 3.19±0.66 |
| Filling degree | Filling degree of water (in the nearest water point) (1-5) | 2.6±0.44 |
| *d* to human | Distance to nearest human population (km) | 11.72±8.02 |
| Ungulate abundance | Total ungulate abundance (estimation of ind/per sampling area)^(1)^ | 789.99±324.69 |
| RA cattle | Relative abundance of cattle respect to the total ungulates per sampling area (%)^(1)^ | 17.84±11.71 |
| RA red deer | Relative abundance of red deer respect to the total ungulates per sampling area (%)^(1)^ | 23.62±16.73 |
| RA fallow deer | Relative abundance of fallow deer respect to the total ungulates per sampling area (%)^(1)^ | 27.30±2.76 |
| RA wild boar | Relative abundance of wild boar respect to the total ungulates per sampling area (%)^(1)^ | 30.96±10.11 |
| P cattle | Proportion of days during which cattle was present in the nearest water point measured by camera trapping (%)^(2)^ | 18.24±11.66 |
| P red deer | Proportion of days during which red deer was present in the nearest water point measured by camera trapping (%)^(2)^ | 65.55±19.87 |
| P fallow deer | Proportion of days during which fallow deer was present in the nearest water point measured by camera trapping (%)^(2)^ | 17.83±11.42 |
| P wild boar | Proportion of days during which wild boar was present in the nearest water point measured by camera trapping (%)^(2)^ | 64.61±9.96 |
| Host spp. | Host species (categorical 1-3) | - |

^(1)^Mammal Monitoring Services in EBD (<http://www-rbd.ebd.csic.es/Seguimiento/seguimiento.htm>).

^(2)^Barasona et al. 2016
